# Amide Proton Transfer-weighted MRI combined with serum prostate-specific antigen levels for differentiating malignant prostate lesions from benign prostate lesions: a retrospective cohort study

**DOI:** 10.1186/s40644-022-00515-w

**Published:** 2023-01-07

**Authors:** Lu Yang, Lei Wang, Yuchuan Tan, Hanli Dan, Peng Xian, Yipeng Zhang, Yong Tan, Meng Lin, Jiuquan Zhang

**Affiliations:** 1 Department of Radiology, Chongqing University Cancer Hospital & Chongqing Cancer Institute & Chongqing Cancer Hospital, No.181 Hanyu Road, Shapingba District, Chongqing, 400030 China; 2 Department of Urology, Chongqing University Cancer Hospital & Chongqing Cancer Institute & Chongqing Cancer Hospital, Chongqing, 400030 China

**Keywords:** Amide proton transfer (APT)-weighted MRI, Prostate-specific antigen, Prostate cancer, Malignant lesions, Benign lesions

## Abstract

**Background:**

Early diagnosis of prostate cancer improves its prognosis, while it is essential to upgrade screening tools. This study aimed to explore the value of a novel functional magnetic resonance imaging (MRI) technique, namely amide proton transfer (APT)-weighted MRI, combined with serum prostate-specific antigen (PSA) levels to differentiate malignant prostate lesions from benign prostate lesions.

**Methods:**

Data of patients who underwent prostate examinations at Chongqing University Cancer Hospital between July 2019 and March 2022 were retrospectively analyzed. All patients underwent T2-weighted imaging (T2WI), APT, diffusion-weighted imaging (DWI), and dynamic contrast-enhanced (DCE) MRI. Two radiologists analyzed the images independently. The ability of the quantitative parameters alone or in different combinations in differentiating malignant prostate lesions from benign prostate lesions were compared by using receiver operating characteristic (ROC) curves. According to the DeLong test, the combined parameters were significantly different from the corresponding single parameter (*P* < 0.05).

**Results:**

A total of 79 patients were finally enrolled, including 52 patients in the malignant group and 27 patients in the benign group. The separate assessment of indexes revealed that APTmax, APTmean, mean apparent diffusion coefficient (ADCmean), ADCmax, ADCmin, tPAD, free prostate-specific antigen (FPSA), FPSA/total prostate-specific antigen (tPSA), and PSA density (PSAD) were significantly different between the two groups (*P* < 0.05), while APTmin was not significantly different between the two groups (*P* > 0.05). APTmax and APTmean had the high values of area under the ROC curve (AUC), which were 0.780 and 0.710, respectively. APTmax had a high sensitivity, and APTmean had a high specificity. The combination of APTmax, APTmean, ADCmean, and PSAD had the highest AUC value (AUC: 0.880, sensitivity: 86.540, specificity: 78.260).

**Conclusion:**

APTmax, APTmean, ADCmean, ADCmin, tPAD, FPSA, and PSAD showed to have a high value in differentiating malignant prostate lesions from benign prostate lesions in the separate assessment of indexes. The combination of APTmax, APTmean, ADCmean, and PSAD had the highest diagnostic value.

## Introduction

Prostate cancer is the second most common cancer in men worldwide and the most common cancer in men in the United States [1, 2]. The annual age-standardized incidence of prostate cancer is 29.3 per 100,000 men for an estimated 1,276,106 cases and 358,989 deaths in 2018 [3]. The incidence of prostate cancer has gradually increased in the recent decades [4]. The median age at the time of diagnosis of prostate cancer is 66 years old. The overall five-year survival rate of patients with prostate cancer is noticeable 98% [5]. However, the five-year survival rate of localized prostate cancer and prostate cancer with extraglandular and distant metastasis is remarkably different. The five-year survival rate for prostate cancer with extraglandular and distant metastasis is 31%. Therefore, the Gleason score and pathological T stage are two of the most important prognostic factors for prostate cancer. Early diagnosis and treatment could improve the quality of life and five-year survival rate of patients with prostate cancer [6].

At present, multiparametric magnetic resonance imaging (mpMRI) is considered as one of the most effective imaging methods in diagnosing prostate cancer. It is noteworthy that MRI has a high soft-tissue resolution and allows the determination of the Prostate Imaging Reporting & Data System (PI-RADS) score [7], differentiation of malignant lesions from benign lesions, pre-operative evaluation of malignant lesions, and evaluation of treatment efficacy [8, 9]. Nevertheless, the sensitivity and specificity of MRI for prostate cancer can still be improved [10, 11]. A recent study explored the diagnostic value of combination of amide proton transfer (APT) and mpMRI in transition zone (TZ) prostate cancer. Differences in APT-weighted and apparent diffusion coefficient (ADC) values between TZ prostate cancer and benign prostatic hyperplasia (BPH), and differences in T2* values between stromal BPH and glandular BPH were found. It was found that APT-weighted and ADC were independent predictors of TZ prostate cancer. Moreover, a combination of APT and ADC values improved the diagnostic sensitivity of TZ prostate cancer and achieved the purpose of improving the diagnostic efficiency [12].

Novel MRI techniques are limited to conventional medical imaging, present the details of morphological features of lesions, and innovatively provide functional parameters, which could be used as imaging biomarkers for diverse types of cancer, thereby facilitating the early diagnosis of malignant lesions. Serum prostate-specific antigen (PSA) is the biochemical biomarker that has been highly acknowledged in clinical practice for prostate diseases [13]. Nevertheless, this biomarker is not perfect [14, 15], and its combination with other biomarkers could result in better sensitivity and specificity for prostate cancer.

It is noteworthy that APT-weighted MRI has advanced rapidly in recent years [16–19]. As an endogenous chemical exchange saturation transfer (CEST) imaging technique and a molecular MRI imaging technique, APT imaging is considered as the most clinically feasible CEST imaging because its specific resonance frequency is different from the water resonance frequency, and it allows the exchange of a large number of water molecules with amide protonic peptides in endogenous mobile proteins to acquire images [16, 17, 19]. APT imaging is based on the fact that tumor cells have a high proliferation activity and a high protein synthesis, leading to differences in protein amounts between benign and malignant lesions [19]. Therefore, acquiring the quantitative APT parameters could quantitatively reflect the molecular changes and could be used as a potential imaging biomarker.

Therefore, the present study aimed to explore the value of the APT-weighted MRI combined with serum PSA levels to differentiate malignant prostate lesions from benign prostate lesions, and to provide more imaging information for early preoperative diagnosis of prostate cancer.

## Methods

### Study design and patients

Data of patients who underwent prostate examinations at Chongqing University Cancer Hospital (Chongqing, China) between July 2019 and March 2022 were retrospectively analyzed. This study was approved by the Ethics Committee of Chongqing University Cancer Hospital. The requirement for patients’ informed consent was waived by the Ethics Committee.

All patients underwent T2-weighted imaging (T2WI), APT, diffusion-weighted imaging (DWI), and dynamic contrast-enhanced (DCE) MRI. The inclusion criteria were as follows: 1) untreated patients with prostate diseases, and 2) patients who received MRI examination. The exclusion criteria were as follows: 1) history of prostate surgery or endocrine therapy, or 2) patients who underwent prostate biopsy within 4–6 weeks before MRI.

### MRI examination

An INGENIA 3.0 T MRI scanner (Philips Healthcare Co., Ltd., Best, The Netherlands) was used for the MRI of prostate of all patients from July 2019 to March 2022, including APT, DWI, T2WI, and DCE, using a 32-channel phased-array body coil (Table 1). The scanning parameters included b = 0 and 1400 s/mm^2^ for DWI and SPIR for fat suppression in APT.

**Table 1:** Parameters of the scanning sequences

Sequences	TR (ms)	TE (ms)	TA (s)	Layers	Layer thickness (mm)	Interlayer spacing (mm)	FOV (mm)	Matrix	NSA	
T2WI	3000	110	1′56’’	24	3	0.3	200 × 200	308 × 255	1	TSE factor 14
DWI	4828	90	3′8’’	24	3	0	200 × 200	80 × 81	1	EPI factor 55
APT	5842	7.9	4′54’’	18	5	0	140 × 140	80 × 78	1	TSE factor 174
T1WI	4.0	2.0	6′24’’	24	3.5	0	250 × 250	180 × 140	1	dynamic phase 80

*TR* repetition time, *TE* echo time, *TA* acquisition time, *FOV* field of view, *NS*A number of signals per acquisition, *T2WI* T2-weighted imaging, *DWI* diffusion-weighted imaging, *APT* amide proton transfer, *T1WI* T1-weighted imaging *TSE* turbo spin echo, *EPI* echo-planar imaging

### Image analysis

**Fig. 1:**
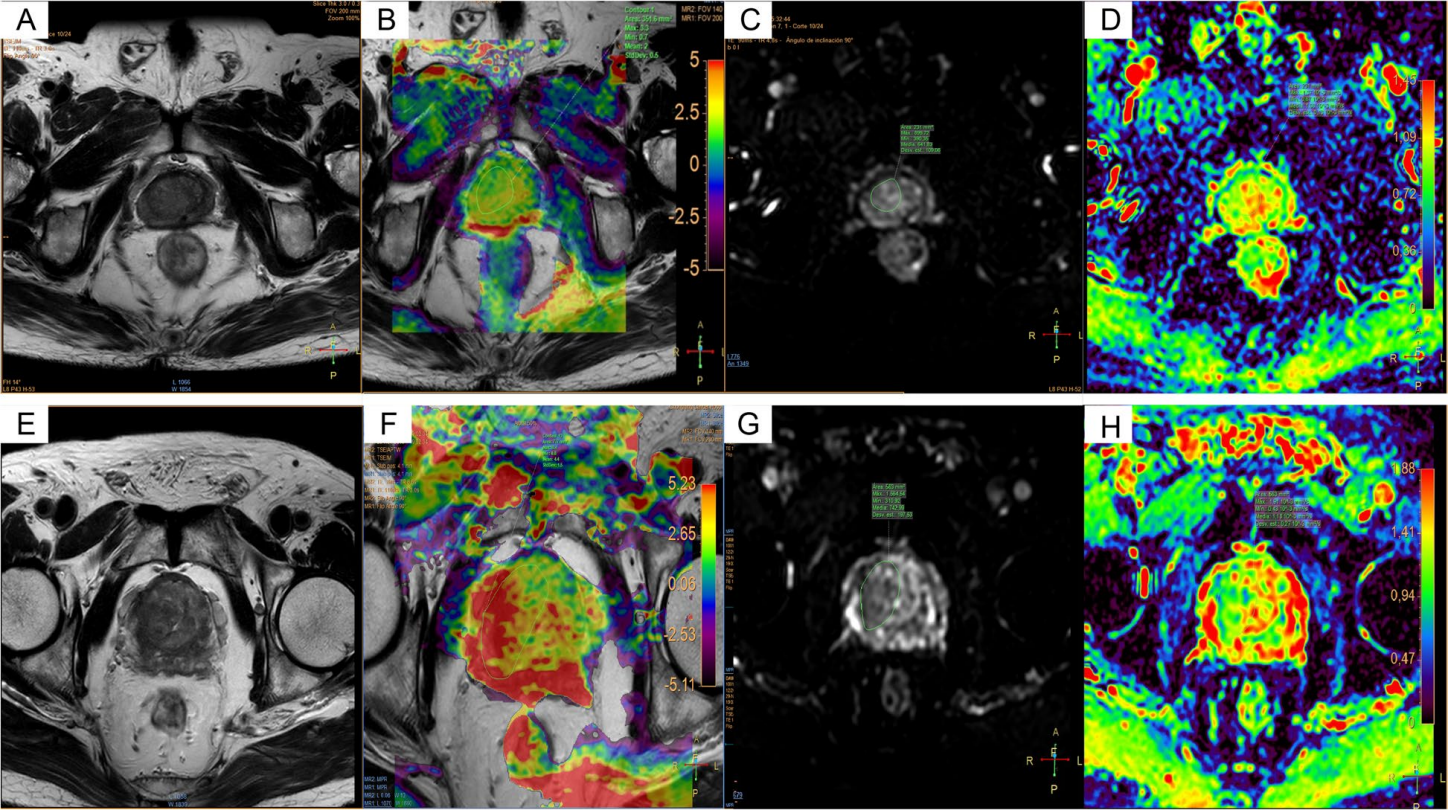
Case examples. 1 A 75-year-old man was hospitalized, and the physical examination revealed that PSA level increased. (1A) T2WI. (1B) APT. (1C) DWI. (1D) ADC. 2 A 68-year-old man was hospitalized for treatment after finding the possibility of prostate malignancy in the external hospital. (2A) T2WI. (2B) APT. (2C) DWI. (2D) ADC

The images were processed by the ISP post-processing workstation (Philips Healthcare Co., Ltd.). Two radiologists reviewed the images independently and blindly. The maximum lesion level was selected using the T2-weighted images as the standard. Then the region of interest (ROI) was selected at the same level on the APT and DWI images to measure the APT and ADC values (Fig. 1). The averages of values measured by the two radiologists were calculated for the statistical analysis. Patients’ medical records were reviewed to obtain the total PSA (tPSA) and free PSA (FPSA).

### Statistical analysis

SPSS 23.0 software (IBM, Armonk, NY, US) was used to perform the statistical analysis. Intraclass correlation (ICC) analysis was performed for the measurement data obtained by the two radiologists (ICC > 0.75 indicated a high consistency, ICC equal to 0.4–0.75 indicated a moderate consistency, and ICC < 0.4 indicated a low consistency). The Kolmogorov–Smirnov test was used to assess the normal distribution, and the Levene’s test was employed for the homogeneity assessment of the continuous data. Continuous data were analyzed using the independent-samples t-test or the Wilcoxon rank-sum test. Two-sided *P* < 0.05 was considered statistically significant. Receiver operating characteristic (ROC) curves were plotted for the analysis. For parameters with statistically significant differences, the logistic regression analysis was used to estimate the probability of the combined parameters. The parameters with statistically significant differences were modeled using the binary logistic regression analysis, and the probability of the combined parameters was calculated. ROC curves were plotted to compare the combinations of parameters in differentiating malignant prostate lesions from benign prostate lesions.

The index with the highest diagnostic accuracy was selected from the separate assessment of APT/ADC and PSA indexes to establish the combined model. The SPSS software was employed to select the binary logistic regression model, and two or more parameters were obtained through modeling, in order to jointly predicting the probability of malignant tumors.

## Results

### General characteristics of patients

A total of 123 patients were recruited, of whom 44 patients were excluded according to the exclusion criteria. Finally, 79 patients were included in this study, of which 52 patients were in the malignant group (pathologically proven with prostate cancer [20]) and 27 patients were in the benign group (pathologically proven with non-cancerous lesions, including prostatic hyperplasia and prostatitis). The mean age was 70.54 ± 8.66 years old. There were no significant differences in age and volume of prostate between malignant group and benign group (*P* < 0.05) (Table 2).

**Table 2:** Characteristics of the patients

Characteristics (*n* = 79)	Malignant (*n* = 52)	Benign (*n* = 27)	*P* value
Age (years)	69.29 ± 8.20	72.19 ± 9.43	0.42
Volume of prostate (cm^3^)	9.34 ± 12.46	10.83 ± 14.66	0.22
PSAD (ng/mL/cm^3^)	69.73 ± 137.59	3.76 ± 7.83	0.00
Gleason score, n (%)			
Low-risk subgroup (≤ 3 + 3 points)	15 (28.85%)		
High-risk subgroup (> 3 + 3 points)	37 (71.15%)		

*PSAD* prostate-specific antigen density

### Consistency between radiologists

ICC consistency was performed for all the measurement data by the two radiologists. As shown in Table 3, APT and ADC parameters showed a moderate consistency between the two radiologists.

**Table 3:** ICC consistency test of the results measured by the two radiologists

	Radiologist 1	Radiologist 2	ICC	95% CI
APTmean (%)	2.44 ± 1.21	2.42 ± 1.38	0.732	0.649–0.789
APTmax (%)	5.56 ± 2.12	6.06 ± 2.26	0.701	0.605–0.768
APTmin (%)	-1.61 ± 2.33	-1.55 ± 2.62	0.652	0.538–0.734
ADCmean (10^–3^ mm^2^/s)	0.91 ± 0.32	0.88 ± 0.37	0.664	0.554–0.743
ADCmax (10^–3^ mm^2^/s)	1.55 ± 0.45	1.47 ± 0.51	0.629	0.507–0.718
ADCmin (10^–3^ mm^2^/s)	0.53 ± 0.34	0.61 ± 0.53	0.566	0.395–0.699

*ICC* intraclass correlation, *CI* confidential interval, *APT* amide proton transfer, *ADC* apparent diffusion coefficient

### Ability of the quantitative parameters in differentiating malignant prostate lesions from benign prostate lesions

**Fig. 2:**
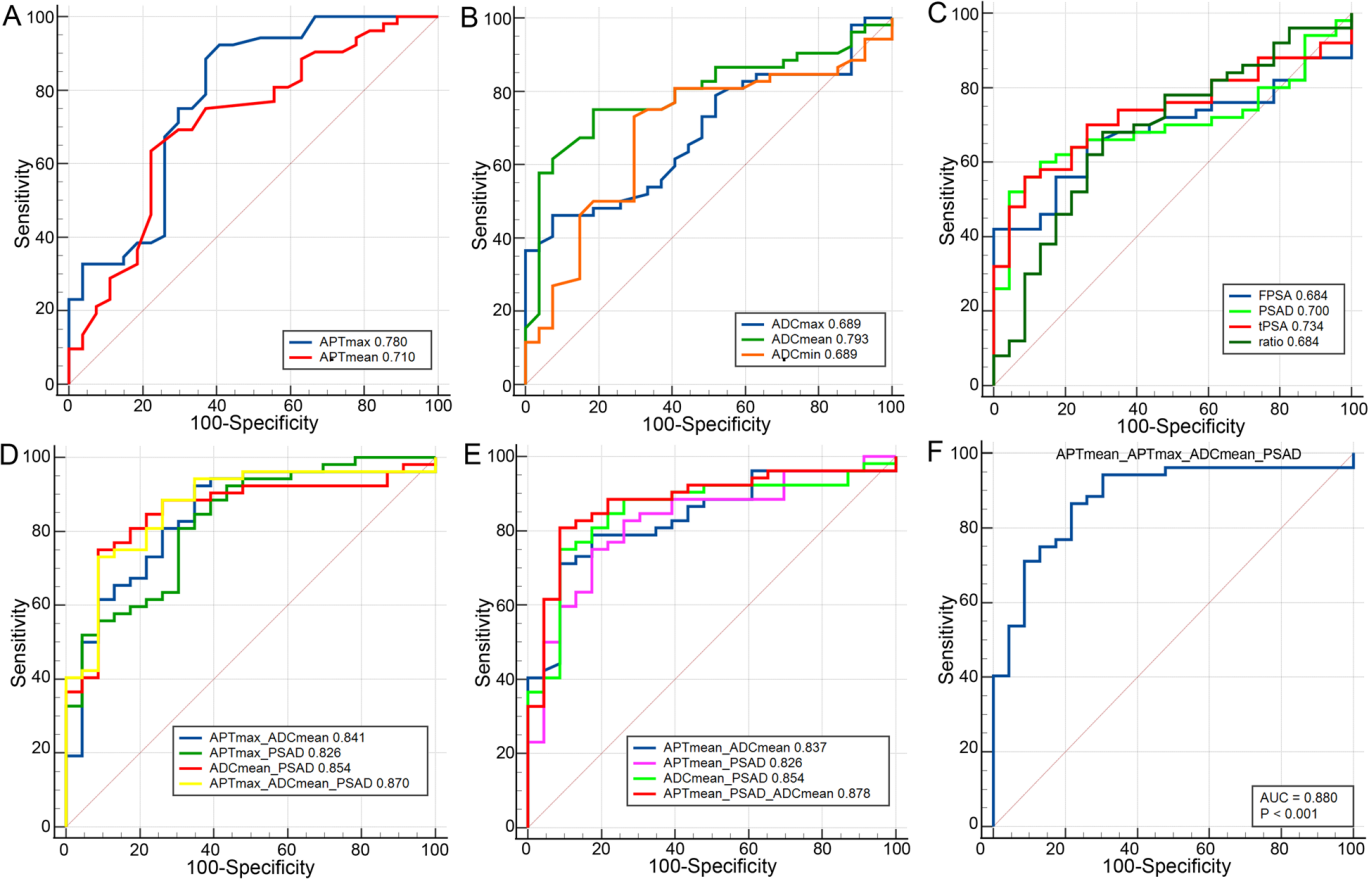
Receiver operating characteristic (ROC) curves. **A** ROC curves of APT parameters. **B** ROC curves of the DWI parameters. **C** ROC curves of the PSA parameters. **D** ROC curves of the combinations of APTmax, ADCmean, and PSAD. **D** ROC curves of the combinations of APTmean, ADCmean, and PSAD. **E** ROC curves of the combinations of APTmax, APTmean, ADCmean, and PSAD. APT: amide proton transfer; ADC: apparent diffusion coefficient; tPSA: total prostate-specific antigen; FPSA: free prostate-specific antigen; ratio: FPSA/tPSA. PSAD: prostate-specific antigen density

As presented in Table 4, the quantitative parameters in differentiating malignant prostate lesions from benign prostate lesions were significantly different (*P* < 0.05), except for APTmin (*P* > 0.05). Quantitative APT parameters, including APTmax and APTmean, could well distinguish malignant prostate lesions from benign prostate lesions. Comparatively, APTmax had a higher sensitivity (92.310), and APTmean had a higher specificity (77.780), while they both had high area under the curve (AUC) values (Fig. 1A and Table 5).The other parameters had relatively high values in differentiating malignant prostate lesions from benign prostate lesions. ROC curves were plotted and showed that ADCmean had the highest AUC value (0.793, 95% confidence interval (CI): 0.688–0.876), APTmax had the highest sensitivity (92.310), and FPSA had the highest specificity (100.000) (Fig. 2A, B, C and Table 5).

**Table 4:** Ability of the quantitative parameters in differentiating malignant from benign prostate diseases

	Malignant (*n* = 52)	Benign (*n* = 27)	Z/t	*P* value
APTmean (%)	2.740 ± 1.106	1.859 ± 1.200	-3.263	0.002
APTmax (%)	6.698 ± 1.837	4.530 ± 1.894	-4.924	< 0.001
APTmin (%)	-1.788 ± 2.557	-1.259 ± 1.815	0.965	0.342
ADCmean (10^–3^ mm^2^/s)	0.845 (0.300–2.030)	1.190 (0.620–1.680)	-4.259	< 0.001
ADCmax (10^–3^ mm^2^/s)	1.450 ± 0.445	1.738 ± 0.391	2.835	0.006
ADCmin (10^–3^ mm^2^/s)	0.464 ± 0.356	0.646 ± 0.271	2.330	0.022
tPSA (ng/mL)	33.604 (0.600–8169.000)	6.053 (0.840–131.760)	-3.321	0.001
FPSA (ng/mL)	3.959 (0.020–483.000)	1.230 (0.210–7.240)	-2.506	0.012
FPSA/tPSA	16.159 ± 15.300	23.336 ± 12.954	2.074	0.043
PSAD (ng/mL/cm^3^)	10.585 (0.010–688.540)	1.980 (0.030–38.650)	-2.884	0.004

*APT* amide proton transfer, *ADC* apparent diffusion coefficient, *tPSA* total prostate-specific antigen, *FPSA* free prostate-specific antigen, *PSAD* prostate-specific antigen density

**Table 5:** Analysis of the ROC curves of the different quantitative parameters

	AUC	95%CI	Sensitivity	Specificity	Youden index J	Best cut-off value	*P* value^a^
APTmax	0.780	0.673–0.865	92.310	59.260	0.516	4.300	< 0.001
APTmean	0.710	0.598–0.807	63.460	77.780	0.412	2.300	0.001
ADCmean	0.793	0.688–0.876	75.000	81.480	0.565	1.060	< 0.001
ADCmax	0.689	0.575–0.788	46.150	92.580	0.388	1.360	0.002
ADCmin	0.689	0.575–0.789	73.080	70.370	0.435	0.620	0.003
tPSA	0.734	0.628–0.836	57.690	91.300	0.490	28.140	< 0.001
FPSA	0.684	0.565–0.788	42.000	100.000	0.420	7.240	0.003
FPSA/tPSA	0.684	0.565–0.788	68.000	69.570	0.376	17.050	0.007
PSAD	0.700	0.594–0.809	53.850	95.650	0.495	8.210	< 0.001

*ROC* receiver operating characteristic, *AUC* area under the curve, *CI* confidence interval, *APT* amide proton transfer, *ADC* apparent diffusion coefficient, *tPSA* total prostate-specific antigen, *FPSA* free prostate-specific antigen, *PSAD* prostate-specific antigen density

^a^DeLong et al., [21]

### Combinations of parameters in differentiating malignant prostate lesions from benign prostate lesions

As reported previously, APTmax, APTmean, and ADCmean [22], the most representative parameters of APT and ADC, were used in combination with PSAD, which is the most representative parameter of PSA [23]. The combinations of these quantitative parameters in differentiating malignant prostate lesions from benign prostate lesions were compared, and the ROC curves were plotted. As illustrated in Fig. 2D, E, F and Table 6, the AUC value was the highest when APTmax, APTmean, and ADCmean were combined with PSAD (AUC: 0.880, 95% CI: 0.784–0.943, sensitivity: 86.540, specificity: 78.260). According to the DeLong test [21], the combined parameters were significantly different from the corresponding single parameter (*P* < 0.05).

**Table 6:** Analysis of the ROC curves of the combinations of different quantitative parameters

	AUC	95%CI	Sensitivity	Specificity	Youden index J	*P* value^a^
APTmax + ADCmean	0.841	0.763–0.928	92.310	70.370	0.627	< 0.001
APTmax + PSAD	0.826	0.721–0.904	80.770	69.570	0.503	< 0.001
ADCmean + PSAD	0.854	0.753–0.925	75.000	91.300	0.663	< 0.001
APTmean + ADCmean	0.837	0.748–0.918	78.850	85.190	0.640	< 0.001
APTmean + PSAD	0.826	0.721–0.904	75.000	82.960	0.576	< 0.001
APTmean + ADCmean + PSAD	0.878	0.782–0.942	80.770	91.300	0.721	< 0.001
APTmax + APTmean + ADCmean + PSAD	0.880	0.784–0.943	86.540	78.260	0.648	< 0.001

*ROC* receiver operating characteristic, *AUC* area under the curve, *CI* confidence interval, *APT* amide proton transfer, *ADC* apparent diffusion coefficient, *tPSA* total prostate-specific antigen, *FPSA* free prostate-specific antigen, *PSAD* prostate-specific antigen density

^a^DeLong et al., [21]

## Discussion

Early diagnosis of prostate cancer improves its prognosis [1, 2], while screening tools should be further upgraded [10, 11, 14, 15]. Therefore, the present study aimed to explore the value of the novel functional MRI technique, APT-weighted MRI, combined with serum PSA levels for differentiating malignant prostate lesions from benign prostate lesions. The results indicated that APTmax, APTmean, ADCmean, ADCmax, ADCmin, tPAD, FPSA, FPSA/tPSA, and PSAD had a high clinical value in differentiating malignant prostate lesions from benign prostate lesions. The combination of APTmax, APTmean, ADCmean, and PSAD showed the highest diagnostic value.

Early diagnosis has important clinical significance for the treatment and prognosis of patients with prostate cancer. Nevertheless, the early differentiation of malignant prostate lesions from benign prostate lesions is still difficult in clinical practice based only on the current imaging methods, such as ultrasound and MRI. The APT imaging technique is based on transferring the amide protons and water, and it reflects the changes of proteins and pH values by variations of water signals. The internal contrast is acquired by measuring the water signals to indirectly acquire the APT-weighted signal values, depending on the exchange ratio between the amide protons and free-water protons. The exchange ratio depends on pH values and protein concentrations in the body. The APT technique was initially used for the nervous system [24–26]. In recent years, a great number of researchers have applied the APT technique to diagnose prostate diseases [27, 28].

The findings of the present study showed that APTmax had a high diagnostic value for differentiating malignant prostate lesions from benign prostate lesions. The sensitivity was the highest, indicating that the maximum transfer of amide protons and exchange ratio of water protons in a lesion could sensitively reflect the occurrence of malignant lesions. Jia et al. [27], for the first time, attempted to apply the APT imaging in prostate diseases, and reported the value of this technique in differentiating malignant prostate lesions from benign prostate lesions. Takayama et al. [28] found that the APT-weighted values in prostate cancer patients with a Gleason score of 7 points were significantly higher than those of patients with other scores. These findings were generally in agreement with our results. The metabolism in malignant prostate lesions is more active than in benign lesions. The exchange ratio of protons is higher, which is consistent with the pathological features of malignant lesions. The differences of APTmax and APTmean, two parameters acquired by APT imaging, were statistically significant. In contrast, the difference of APTmin was not statistically significant, which could be associated with the fact that APTmin expresses the lowest value of protein content in the region of interest, and the difference is not enough to distinguish between benign and malignant lesions. Importantly, the minimum exchange ratio of protons could not reflect the degree of metabolic activity.

The results of the present study revealed that the differences of APTmax, APTmean, ADCmean, ADCmax, ADCmin, tPAD, FPSA, FPSA/tPSA, and PSAD were statistically significant (*P* < 0.05), suggesting that these parameters had high diagnostic values in differentiating malignant prostate lesions from benign prostate lesions. Among these parameters, ADCmean had the highest AUC, APTmax had the highest sensitivity, and FPSA had the highest specificity and the lowest sensitivity, reflecting that FPSA had the highest diagnostic accuracy and a relatively high false-negative rate. This indicated that functional MRI sequences, such as DWI and PSA, can be used as independent predictive biomarkers to discriminate benign prostate lesions and malignant prostate lesions. In contrast, APTmax had the highest positive rate. After combining these parameters, the results showed that the combination of APTmax, APTmean, ADCmean, and PSAD had the highest AUC and sensitivity, suggesting that this combination had the highest diagnostic value for prostate cancer. Compared with using PSA and DWI alone, the combination of APT, DWI, and PSA techniques had a relatively high diagnostic value (AUC: 0.880) and a high sensitivity (86.540) for prostate cancer. A previous study demonstrated that the AUC values for the biparametric MRI (bpMRI) and multiparametric MRI (mpMRI) protocols for prostate cancer were comparable (0.790 [0.732–0.840] and 0.791 [0.733–0.841], respectively) [29]. Guo et al. [12] found that APTmean and ADC were independent predictors of TZ prostate cancer. Moreover, combination of APTmean and ADC values improved the sensitivity of the diagnosis of TZ prostate cancer and achieved the purpose of improving the diagnostic efficiency, which are similar to the results of the present study.

The advantage of APT-weighted MRI is that it is a 3D imaging technique. Compared with the conventional two-dimensional (2D) APT technique [30], this technique could scan multiple layers in a short time, acquire the APT image of the whole prostate region, and provide more comprehensive functional information.

There were several limitations in the present study. First, the sample size was small and imbalance, and additional studies are required to determine the exact diagnostic value of APT for prostate cancer. Second, this was a single-center study, and local practice biases could influence the results. Multicenter studies can not only increase the sample size, but also mitigate the risk of bias. Last but not least, because of the small sample size, no direct comparison was performed among imaging techniques.

## Conclusions

APT has a diagnostic value for prostate cancer. APTmax, APTmean, ADCmean, ADCmin, tPAD, FPSA, and PSAD showed to have a high diagnostic value in differentiating malignant prostate lesions from benign prostate lesions. The combination of APTmax, APTmean, ADCmean, and PSAD had the highest diagnostic value.

## Data Availability

All data generated or analyzed during this study are included in this published article.

## Abbreviations

ADC: Apparent diffusion coefficient
APT: Amide proton transfer
AUC: Area under the curve
CEST: Chemical exchange saturation transfer
DWI: Diffusion-weighted imaging
FPSA: Free prostate-specific antigen
ICC: Intraclass correlation
MRI: Magnetic resonance imaging
PI-RADS: Prostate Imaging Reporting & Data System
PSA: Prostate-specific antigen
ROC: Receiver operating characteristic
ROI: Region of interest
DCE: T1-weighted dynamic contrast-enhanced (DCE)
T2WI: T2-weighted imaging
tPSA: Total prostate-specific antigen

## References

[1] Mottet N, Corford P, van den Bergh RCN, Briers E, De Santis M, Fanti S (2020). European Association of Urology (EAU).

[2] NCCN Clinical Practice Guidelines in Oncology (NCCN Guidelines) (2020). Prostate Cancer. Version 3.2020.

[3] Bray F, Ferlay J, Soerjomataram I, Siegel RL, Torre LA, Jemal A (2018). Global cancer statistics 2018: GLOBOCAN estimates of incidence and mortality worldwide for 36 cancers in 185 countries. CA Cancer J Clin.

[4] Jemal A, Bray F, Center MM, Ferlay J, Ward E, Forman D (2011). Global cancer statistics. CA Cancer J Clin.

[5] Siegel RL, Miller KD, Jemal A (2020). Cancer statistics, 2020. CA Cancer J Clin.

[6] Nyame YA, Gore JL (2020). What goes up must come down: identifying truth from global prostate cancer epidemiology. Eur Urol.

[7] PI-RADS (2019). Prostate Imaging - Reporting and Data System Version 21.

[8] Winkel DJ, Wetterauer C, Matthias MO, Lou B, Shi B, Kamen A (2020). Autonomous detection and classification of PI-RADS lesions in an MRI screening population incorporating multicenter-labeled deep learning and biparametric imaging: proof of concept. Diagnostics (Basel)..

[9] Qi Z, Li W, Tan J, Wang C, Lin H, Zhou B (2019). Effect of ginsenoside Rh(2) on renal apoptosis in cisplatin-induced nephrotoxicity in vivo. Phytomedicine.

[10] Zhen L, Liu X, Yegang C, Yongjiao Y, Yawei X, Jiaqi K (2019). Accuracy of multiparametric magnetic resonance imaging for diagnosing prostate Cancer: a systematic review and meta-analysis. BMC Cancer.

[11] Becker AS, Kirchner J, Sartoretti T, Ghafoor S, Woo S, Suh CH (2020). Interactive, up-to-date meta-analysis of MRI in the management of men with suspected prostate cancer. J Digit Imaging.

[12] Guo Z, Qin X, Mu R, Lv J, Meng Z, Zheng W (2022). Amide proton transfer could provide more accurate lesion characterization in the transition zone of the prostate. J Magn Reson Imaging.

[13] Pezaro C, Woo HH, Davis ID (2014). Prostate cancer: measuring PSA. Intern Med J.

[14] Ilic D, Djulbegovic M, Jung JH, Hwang EC, Zhou Q, Cleves A (2018). Prostate cancer screening with prostate-specific antigen (PSA) test: a systematic review and meta-analysis. BMJ.

[15] Lee SK, Kim J-Y, Jeong HS (2020). Benign peripheral nerve sheath tumor of digit versus major-nerve: comparison of MRI findings. PLoS ONE.

[16] Meng N, Wang X, Sun J, Han D, Ma X, Wang K (2020). Application of the amide proton transfer-weighted imaging and diffusion kurtosis imaging in the study of cervical cancer. Eur Radiol.

[17] Kamitani T, Sagiyama K, Togao O, Yamasaki Y, Hida T, Matsuura Y (2020). Amide proton transfer (APT) imaging of parotid tumors: Differentiation of malignant and benign tumors. Eur J Radiol.

[18] Ma X, Bai Y, Lin Y, Hong X, Liu T, Ma L (2017). Amide proton transfer magnetic resonance imaging in detecting intracranial hemorrhage at different stages: a comparative study with susceptibility weighted imaging. Sci Rep.

[19] Sartoretti T, Sartoretti E, Wyss M, Schwenk A, Najafi A, Binkert C (2019). Amide proton transfer contrast distribution in different brain regions in young healthy subjects. Front Neurosci.

[20] Humphrey PA, Moch H, Cubilla AL, Ulbright TM, Reuter VE. The

[21] DeLong, E, DeLong, D, Clarke-Pearson, D. Comparing the Areas under Two or More Correlated Receiver Operating Characteristic Curves: A Nonparametric Approach BIOMETRICS. 1988;44(3):837. 3203132

[22] Saito S, Koyama Y, Ueda J, Hashido T. Relationship between apparent diffusion coefficient distribution and cancer grade in prostate cancer and benign prostatic hyperplasia. Diagnostics (Basel). 2022;12(2):525. 10.3390/diagnostics12020525 PMC8871382 35204614

[23] Milkovic B, Dzamic Z, Pejcic T, Kajmakovic B, Nikolic D, Cirovic D, et al. Evaluation of free-to-total prostate specific antigen (F/T PSA), prostate specific antigen density (PSAD) and (F/T)/PSAD sensitivity on reduction of unnecessary prostate biopsies for patients with PSA in gray zone. Ann Ital Chir. 2014;85(5):448–53. 25599711

[24] Kang XW, Xi YB, Liu TT, Wang N, Zhu YQ, Wang XR, et al. Grading of Glioma: combined diagnostic value of amide proton transfer weighted, arterial spin labeling and diffusion weighted magnetic resonance imaging. BMC Med Imaging. 2020;20(1):50. 10.1186/s12880-020-00450-x PMC7227252 32408867

[25] Zhang Z, Zhang C, Yao J, Chen X, Gao F, Jiang S, et al. Protein-based amide proton transfer-weighted MR imaging of amnestic mild cognitive impairment. Neuroimage Clin. 2020;25:102153. 10.1016/j.nicl.2019.102153 PMC6948365 31901792

[26] Zhou J, Heo HY, Knutsson L, van Zijl PCM, Jiang S. APT-weighted MRI: Techniques, current neuro applications, and challenging issues. J Magn Reson Imaging. 2019;50(2):347–64. 10.1002/jmri.26645 PMC6625919 30663162

[27] Jia G, Abaza R, Williams JD, Zynger DL, Zhou J, Shah ZK, et al. Amide proton transfer MR imaging of prostate cancer: a preliminary study. J Magn Reson Imaging. 2011;33(3):647–54. 10.1002/jmri.22480 PMC4287206 21563248

[28] Takayama Y, Nishie A, Sugimoto M, Togao O, Asayama Y, Ishigami K, et al. Amide proton transfer (APT) magnetic resonance imaging of prostate cancer: comparison with Gleason scores. MAGMA. 2016;29(4):671–9. 10.1007/s10334-016-0537-4 26965511

[29] Xu L, Zhang G, Shi B, Liu Y, Zou T, Yan W, et al. Comparison of biparametric and multiparametric MRI in the diagnosis of prostate cancer. Cancer Imaging. 2019;19(1):90. 10.1186/s40644-019-0274-9 PMC6925429 31864408

[30] Nishie A, Takayama Y, Asayama Y, Ishigami K, Ushijima Y, Okamoto D, et al. Amide proton transfer imaging can predict tumor grade in rectal cancer. Magn Reson Imaging. 2018;51:96–103. 10.1016/j.mri.2018.04.017 29729438

